# Administration of vitamin E attenuates airway inflammation through restoration of Nrf2 in a mouse model of asthma

**DOI:** 10.1111/jcmm.16675

**Published:** 2021-06-04

**Authors:** Quang Luu Quoc, Tra Cao Thi Bich, Seo‐Hee Kim, Hae‐Sim Park, Yoo Seob Shin

**Affiliations:** ^1^ Department of Allergy and Clinical Immunology Ajou University School of Medicine Suwon South Korea

**Keywords:** asthma, Nrf2, ROS, vitamin E

## Abstract

Accumulating evidence reveals that ROS is one of the key mediators that contribute to the development of asthma. Studies on antioxidants have shown to have beneficial effects on asthma management. However, we still do not know the precise mechanism, and the effects depend on age. This study was conducted to assess the levels of ROS and the effect of antioxidants in younger and older mice using an eosinophilic asthma model. We analyzed airway hyperresponsiveness (AHR), cytokines in bronchoalveolar lavage fluid (BALF), inflammatory cell counts, and the expression levels of NFκB, Nrf2, EPx, and EDN in the lung tissue, as well as the level of ROS in the lung tissue and BALF. The degree of eosinophilia and the levels of IL‐5, ROS, and NFκB were significantly increased, whereas the endogenous levels of vitamin E and Nrf2 were decreased in the lung and BALF in the older mice compared to younger mice. The administration of vitamin E attenuated AHR, airway inflammation, and the level of IL‐13 and ROS and enhanced the Nrf2 level in the older mice compared to the younger mice. Taken together, vitamin E treatment may have the therapeutic potential through restoration of the Nrf2 level, especially in elderly asthma.

## 1 INTRODUCTION

Asthma is a chronic inflammatory lung disease characterized by airway hyperresponsiveness, inflammation, and remodeling. The prevalence of asthma has continuously increased in the last decade.^1^, ^2^ In particular, the prevalence of asthma among the elderly increases as life expectancy becomes longer.^3^ The higher rates of hospitalization, morbidity, and mortality in elderly asthmatic patients lead to an unmet medical need and a burden on society.^3^ Moreover, elderly asthmatic patients are often refractory to the existing treatment options because of their corticosteroid resistance, non‐type 2 phenotype, and a low prevalence of atopy.^3^, ^4^

Reactive oxidative species (ROS) play an essential role in maintaining and protecting against endogenous and exogenous pathogens and tend to increase with age.^2^, ^5^ However, ROS overproduction can lead to DNA damage through oxidation of nucleobases, alteration in protein function, or reduction in protein oxidation.^6^ Consequently, ROS cause changes in signaling output, enzyme activity, gene transcription, as well as membrane and genome integrity.^7^ Increased ROS also influence inflammatory diseases.^8^ For example, increased ROS activate pyrin domain‐containing 3 inflammasomes in autoimmune encephalomyelitis.^9^ Cellular apoptosis induced by excessive ROS production can contribute to the development of inflammatory bowel disease.^10^

Immune cells, such as eosinophils and neutrophils, are the main source of ROS, and ROS levels are positively correlated with the severity of asthma and the age of onset of asthma.^2^, ^11^ In addition, recent studies have shown that increased ROS can contribute to tissue damage in the asthma airways by releasing eosinophil extracellular traps.^12^, ^13^

The antioxidant system consists of endogenous and exogenous parts. Endogenous enzymatic antioxidants, including superoxide dismutase (SOD), glutathione peroxidase, and catalase (CAT), have the neutralizing capacity on the degree of ROS production.^14^ This system is controlled by nuclear factor erythroid 2‐related factor 2 (Nrf2). Under normal conditions, Nrf2 was inactivated by binding natural inhibitors called Kelch‐like ECH‐associated protein 1 (Keap1) in the cytoplasm. Nevertheless, stress‐activated Nrf2 can then translocate to the nucleus and interact with HO‐1 and antioxidant response element (ARE).^15^ As a consequence, the Nrf2‐HO1‐Keap1 axis can regulate redox signaling, proteostasis, DNA repair, apoptosis prevention, and iron and heme metabolism.^14^ Nrf2 is also known as an age‐related factor.^16^

Antioxidants are also supplied by reducing compounds, such as vitamin C, vitamin E, carotenoids, and polyphenols, which play integral roles in various antioxidant mechanisms.^17^ Vitamin E is a group of potent, lipid‐soluble, chain‐breaking antioxidants that can prevent oxidative stress.^8^ It consists of multiple natural and unsaturated isoforms, such as α‐, β‐, γ‐, and δ‐tocopherols, which differ in the number of methyl groups on a chromanol head.^8^ The administration of vitamin E decreases C‐reactive protein and pro‐inflammatory cytokine release in coronary artery disease.^18^ Additionally, vitamin E exerts anti‐inflammatory and anticarcinogenic activities by reducing the inflammation index and the number of adenomas in the colon of azoxymethane‐administered mice.^19^ Previous studies reported that the *α*‐tocopherol isoform has been measured to be low in asthmatic patients, and the treatment with this isoform has beneficial effects on the outcome of asthmatic patients.^20^ The treatment of the *α*‐tocopherol isoform attenuates eosinophil recruitment and blocks AHR through direct regulation of endothelial cell signals during leukocyte recruitment in an OVA asthma model.^21^ However, mechanisms underlying effects of vitamin E in younger and older asthmatic patients have not been completely understood yet. Thus, this study was conducted to evaluate the antioxidant–oxidant defense mechanism in older asthmatic mice compared to younger asthmatic mice.

## 2 MATERIALS AND METHODS

### 2.1 Animals

Six weeks‐old BALB/c mice purchased from Jackson Laboratory (Bar Harbor, ME) were used as the younger group. For the older group, 6 weeks‐old mice were bred to the age of 24 weeks under specific pathogen‐ and ovalbumin (OVA)‐free conditions and were housed in a 12 hour light–dark cycle with food and water ad libitum. All animal experiments performed in this study were approved by the Institutional Animal Care and Use Committee of Ajou University (IACUC 2018‐0041).

### 2.2 Allergen sensitization, challenge, and drug treatment protocol

Mice were sensitized intraperitoneally with 10 µg of OVA (Fisher Scientific) in 1 mg of alum (Inject Alum; Pierce) on days 0 and 14, as previously described.^22^ On days 28, 29, and 30, mice were subjected to airway allergen challenges by nebulization with 1% OVA for 30 minutes, using an ultrasonic nebulizer (NE‐Y2, Omron). In some experiments, mice were given vitamin E (Sigma‐Aldrich) (50 mg/kg) orally 30 minutes before OVA challenge, and the last doses were given from days 28 to 32. Control animals received PBS alone. On day 32, 30 minutes after the last vitamin E treatment, mice were euthanized for further analyses. The scheme of this study is shown in Figure S1.

### 2.3 Evaluation of airway resistance, cytokines in bronchoalveolar lavage fluid (BALF), and lung histology

Mice were anesthetized with pentobarbital sodium. The trachea was exposed, and a cannula was inserted. It was connected to a FlexiVent system (Scireq). Then, ventilation was instituted with a tidal volume of 10 ml/kg at a frequency of 150 breaths/minutes. Airway hyperresponsiveness (AHR) to acetyl‐β‐methyl choline chloride was recorded at methacholine doses of 0, 1.56, 3.12, 6.25, 12.5, and 25 mg/ml PBS. BALF was collected by washing with 1X phosphate‐buffered saline (PBS) plus 1% bovine serum albumin (Sigma‐Aldrich) through a cannula. Then, the BALF was centrifuged at 1200 revolutions per minute for 5 minutes at 4°C, and the supernatant was harvested and stored at −70°C until measurement of cytokine levels. Total and differential cell counts were determined by using a hemocytometer and analyzed by using ImageJ (National Institutes of Health, Bethesda). Consecutively, lung tissues were perfused as follows: the left lungs were fixed in 4% paraformaldehyde, while the right lungs were used to measure ROS levels and the expression of various proteins. The fixed tissues were embedded in paraffin and sectioned at 5 μm thickness. To detect inflammatory cells, the sections were stained with hematoxylin and eosin (H&E), and mucus‐containing cells were stained with periodic acid–Schiff (PAS).

### 2.4 Measurement of intracellular ROS

Intracellular ROS were measured as previously described.^23^ BALF cell pellets and lung tissue‐derived cells were washed with 1X PBS. Then, 2 × 10^5^ cells/200µl was incubated with 20 µmol/L H2DCFDA (Molecular Probes, Eugene) for 30 minutes. H2DCFDA‐labeled ROS were detected at dichlorofluorescein intensity by flow cytometry using 488 nm laser for excitation and detected at 535 nm.

### 2.5 Measurement of inflammatory cytokines

The levels of inflammatory cytokines in the serum and BALF were measured by enzyme‐linked immunosorbent assay (ELISA) according to the manufacturer's protocol. ELISA kits for IFN‐γ and IL‐10 were purchased from R&D Systems. The IL‐5 and IL‐13 ELISA kits were purchased from Thermo Fisher Scientific. GSH was purchased from Cayman Chemical Co. For mouse serum, a superoxide dismutase (SOD) assay kit was purchased from Cayman Chemical Co, and the vitamin E ELISA kit was purchased from MyBioSource Inc.

### 2.6 Detection of eosinophil activation and oxidative stress by Western blot

Proteins were isolated from the right lung tissues by lung homogenates, followed by incubation with lysis buffer. Aliquots of 50 μg of total protein per well were loaded onto 12% sodium dodecyl sulfate–polyacrylamide gel and transferred to a polyvinylidene difluoride membrane (Bio‐Rad, Hercules). After blocking in 5% bovine serum albumin (BSA) or skim milk (Sigma Aldrich) in Tris‐buffered saline containing 0.1% Tween 20 (TBS‐T) for 1 hour at room temperature, the membranes were incubated with primary antibodies against EPx, p‐NFκB, NFκB (Santa Cruz Biotechnology), Nrf2, and EDN (Abcam, Cambridge, UK) overnight at 4°C with gentle shaking. Then, the membranes were washed 3 times with TBS‐T for 10 minutes each and incubated with horseradish peroxidase‐conjugated with anti‐goat or anti‐rabbit antibody for 1 hour at room temperature. Signals were detected using ECL Plus Western Blotting Detection Reagents (GE Healthcare). The intensity of the bands was analyzed using a gel doc system (Bio‐Rad Laboratories, Inc, Hercules).

### 2.7 Analysis of NFκB p65 and EPx by immunofluorescence in lung tissues

Analysis of the immunocontent of NFκB p65 was performed in formalin‐fixed lungs by immunofluorescence. First, the lungs were embedded in paraffin, and histological sections of 5 μm were made. The slices were deparaffinated in xylene and rehydrated in ethanol in a concentration‐dependent manner. The slides were boiled in a microwave in sodium citrate for 2 minutes for antigen retrieval. Then, the slices were incubated with 0.1% Triton X for 10 minutes and blocked with 5% BSA in 10% normal donkey serum at room temperature for 1 hour. The sections were incubated overnight with anti‐NF‐kB p65 (1:200) and anti‐EPx (1:200), followed by Alexa Fluor 488 donkey anti‐rabbit and 594 donkey anti‐goat (1:200, Thermo Life Technology, Waltham, MA) for 1 hour. The slices were incubated with DAPI (1:1.000) for 5 minutes. Images were taken under a Zeiss Confocal Laser Scanning Microscope.

### 2.8 Eosinophil isolation

Eosinophils were isolated from healthy controls using the Eosinophil Isolation Kit (Miltenyi Biotec Inc). Briefly, blood was layered on the Lymphoprep (Axis‐Shield), followed by density gradient centrifugation at 2000 rpm at 20°C for 25 minutes without brakes. The granulocyte fraction was lysed for red blood cells by using cold distilled water for 30 seconds. We collected unlabeled cells that pass through. Cell purity (>5%) was evaluated by flow cytometry based on Siglec‐8 and ECP expression for eosinophils, and cell viability evaluated after vitamin E treatment was more than 90% at 800 μmol/L (data not shown).

### 2.9 H_2_O_2_ measurement

Cells were pretreated with vitamin E (8, 80, 800 μmol/L) in serum‐free media for 1 hour. Then, cells were primed with IL‐5 for 30 minutes and LPS for 24 hours. An Amplex™ Red Hydrogen Peroxide/Peroxidase assay kit was purchased from Thermo Fisher Scientific according to the manufacturer's protocols.

### 2.10 ROS measurement for eosinophils

Cells were pretreated with vitamin E (8, 80, 800 μmol/L) in serum‐free media for 1 hour. Eosinophils were pretreated with vitamin E (8, 80 μmol/L) in serum‐free media for 1 hour. Then, cells were stimulated with H_2_O_2_ in a time‐and dose‐dependent manner in order to measure the levels of ROS. Cells were labeled with H2DCDA within 30 minutes and analyzed by using FACS.

### 2.11 Eosinophil treatment protocol

Eosinophils were stimulated with 25 μmol/L H_2_O_2_ in a time‐dependent manner (6, 12, 24 hours) for the expressions of Nrf2 and HO‐1. In some experiments, cells were pretreated with 8, 80, and 800 μmol/L vitamin E within 1 hour and stimulated with 25 μmol/L H_2_O_2_ within 24 hours for ICC and Western blot. For ICC, 2x10^5^ cells were seeded on the poly‐L‐lysine‐coated glasses and treated following the previous protocol. After that, the cells were incubated with anti‐NF‐kB p65 (1:200) and anti‐Nrf2 (1:200) overnight. The cells were analyzed by a Zeiss Confocal Laser Scanning Microscope.

### 2.12 Statistical analysis

Data are presented as mean ± SEM. The data obtained were analyzed using one‐way ANOVA followed by Turkey's *post hoc* test for multiple comparisons of data. A nonparametric *t* test was done to examine the difference between 2 groups. Differences were considered significant at *P* < .05. Data analysis and graph preparation were performed by using the statistical software packages IBM SPSS 20.0 and GraphPad Prism 6 respectively.

## 3 RESULTS

### 3.1 Different features in younger and older asthmatic mice

**FIGURE 1:**
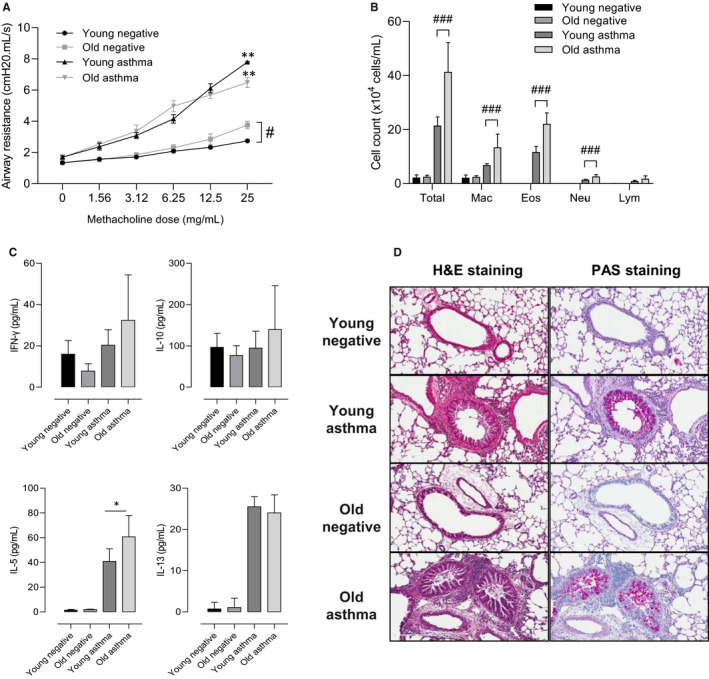
Effects of age on airway hyperresponsiveness and inflammation. Mice were assessed for AHR to methacholine at different concentrations (0, 1.56, 3.12, 6.25, 12.5, and 25 mg/ml) (A). Total and differential cell counts in BALF (B). The level of cytokines released in BALF (C). Lung tissue histology with H&E and PAS staining (D). Data are presented as means ± SD. *P < .05, **P < .01, ***P < .001 obtained by Student's *t* test. BALF, bronchoalveolar lavage fluid; Eos, eosinophils; H&E, hematoxylin and eosin; IFNγ, interferon gamma; IL, interleukin; Mac, macrophages; NC, normal control; Neu, neutrophils; PAS, periodic acid–Schiff

The OVA nebulization in OVA‐sensitized mice led to significantly higher AHR and inflammatory cell numbers in BALF in both younger and older mice (*P* < .050 for all, Figure 1A‐B). Although the degree of AHR was not significantly higher in the older mice than in the younger mice, the inflammatory cell counts, especially eosinophils and neutrophils, increased with age (*P* < .010). There was a significant increase in the level of IL‐5, but not of IL‐13, IFN‐γ, and IL‐10 with age (Figure 1C). Lung histology revealed increased inflammatory cells and mucus secretion in the older mice compared to the younger mice (Figure 1D).

### 3.2 Oxidant stress and inflammatory markers in the younger and older asthmatic mice

**FIGURE 2:**
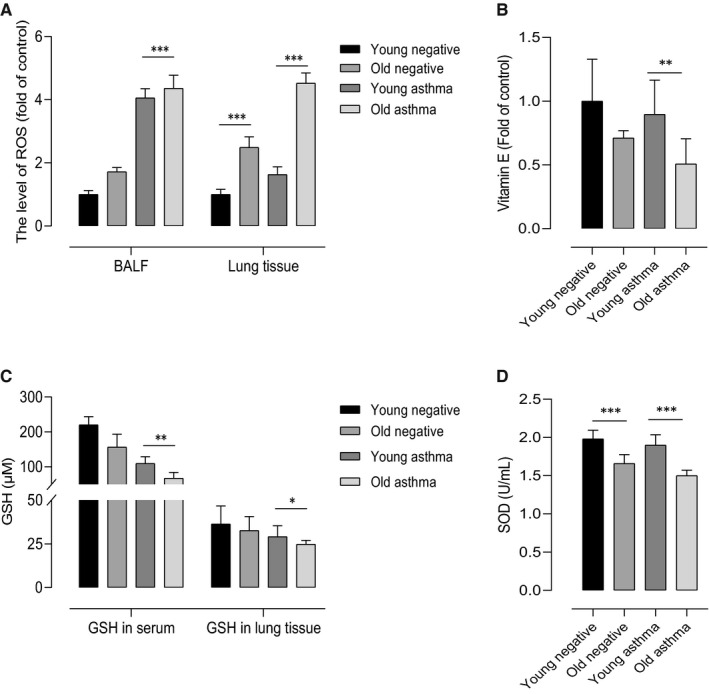
Effects of age on oxidant–antioxidant defense. ROS in the BALF and lung tissues (A) was higher, whereas the vitamin E level in serum (B) was lower in the old mice than young mice. The levels of GSH (C) in serum and lung tissues, and SOD (D) were decreased in the serum of the old mice compared to the young mice. Data are presented as ± SD. *P < .05, **P < .01, ***P < .001 obtained by Student's *t* test. BALF, bronchoalveolar lavage fluid; GSH, glutathione; ROS, reactive oxygen species; SOD, superoxide dismutase

Oxidative stress and endogenous antioxidant levels were measured in the younger and older asthmatic mice. There was a significant increase in the levels of ROS in BALF and lung tissues (*P* < .010, Figure 2A), whereas the level of vitamin E in serum (*P* < .010), GSH in BALF and lung tissues (*P* = .004, *P* < .050, respectively), and total SOD activity in serum (*P* < .001) were reduced in the older asthmatic mice compared to the younger asthmatic mice (Figure 2B‐D).

**FIGURE 3:**
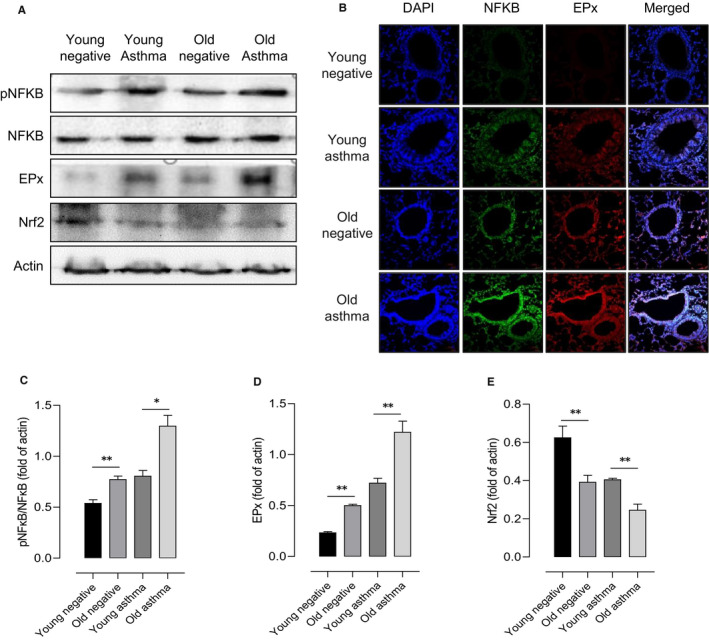
Oxidative stress and inflammatory markers in lung tissue with aging. Western blot determined EPx, NFKB, and Nrf2 expressions in lung tissue homogenates of young and old mice after saline or OVA challenge. Then, β‐actin was used as a loading control (A). Analysis of the NFκB p65 protein and EPx by immunofluorescence in lung sections (B). Quantitative data of NF‐κB, EPx (C, D) and Nrf2 (E). *P < .05, **P < .01, ***P < .001 obtained by Student's *t* test. EPx, eosinophil peroxidase; Nrf2, nuclear factor erythroid 2‐related factor 2; NFKB, nuclear factor kappa B

Next, pro‐inflammatory markers including eosinophil activation were measured in lung homogenates. The expressions of NFκB and EPx were increased in the older asthmatic mice compared to the younger mice by Western blot (Figure 3A). Signals for NFκB were enhanced in the younger and older asthmatic mice compared to the negative control mice in the immunofluorescence study. It was also showed that the expressions of EPx and NFκB were increased in the older mice compared to the younger mice (Figure 3B). Interestingly, we also found the significant decrease level of Nrf2 in the older asthmatic mice. These findings suggest that there is a significant increase in oxidative stress, causing eosinophilic activation, especially in the older asthmatic mice.

### 3.3 The effect of vitamin E treatment on AHR and airway inflammation in the younger and older asthmatic mice

**FIGURE 4:**
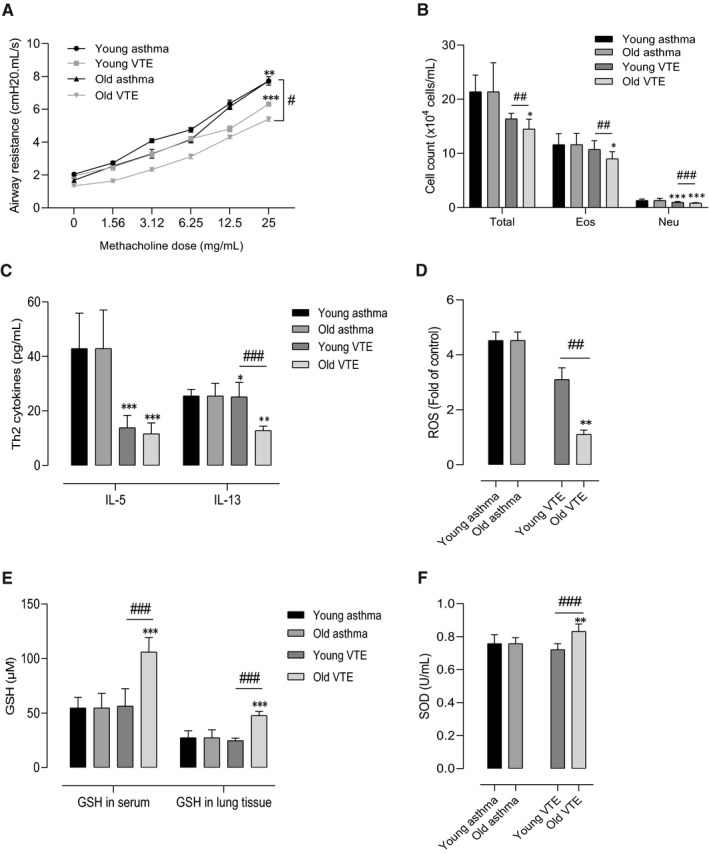
Effect of vitamin E treatment on airway hyperresponsiveness and pulmonary inflammation with aging. Vitamin E decreased the AHR (A). The cell counts in BALF (B). Vitamin E decreased the level of Th2 cytokines (C) and ROS formation (D). Vitamin E restored the GSH level in serum and lung tissues (E) as well as the SOD level in serum (F). ^***^ *P* < .001 at the respective weeks, between the positive asthma groups (young negative, old negative) *vs*. the treatment groups (young asthma, old asthma); ^###^ *P* < .001 between the treatment groups at 6 and 24 w. Data were normalized in order to compare between the effects of antioxidant treatment on the asthma mouse model

To determine the effects of vitamin E treatment on OVA‐induced AHR and airway inflammation, the mice were treated with 50 mg/kg vitamin E during the OVA challenge and continued 2 days more in both younger and older asthmatic mice. Vitamin E treatment significantly reduced AHR and the inflammatory cell counts, such as eosinophils and neutrophils, in the younger and older asthmatic mice (*P* < .050 for all, Figure 4A,B), which was more prominent in the older asthmatic mice than in the younger asthmatic mice. Vitamin E treatment also considerably decreased the release of IL‐5 and IL‐13 in BALF (*P* < .010 for all, Figure 4C) and the production of ROS in the lung tissues (*P* < .001, Figure 4D); it also restored the serum activity of SOD and GSH in serum and lung tissues, especially in the older asthmatic mice (*P* < .001, Figure 4E‐F).

### 3.4 The effect of vitamin E treatment on Nrf2 and NF‐κB expressions and its cascade

**FIGURE 5:**
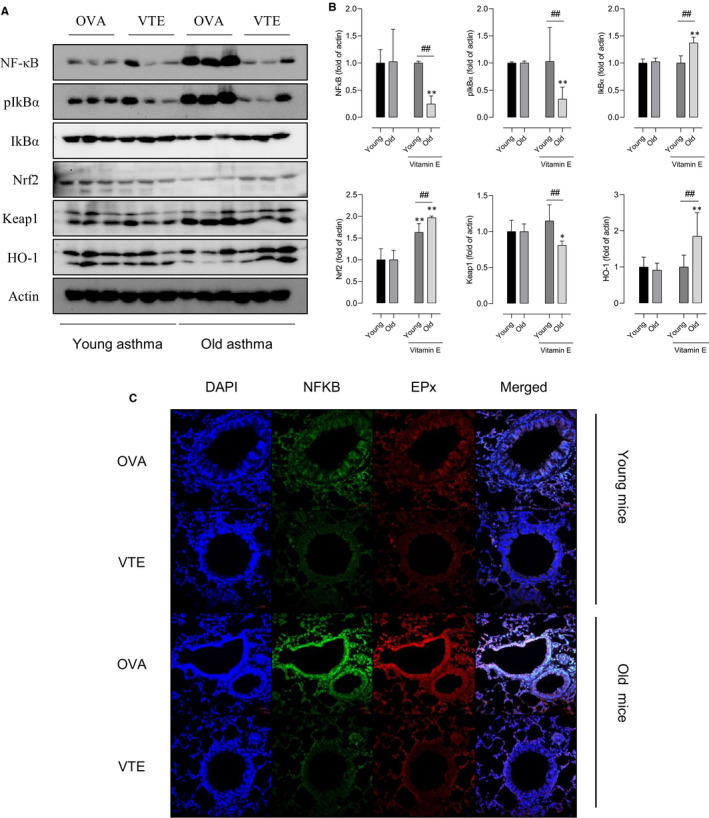
Effect of vitamin E treatment on Nrf2 and NF‐kB expression. Expressions of NFκB, IkBα, Nrf2, Keap1, and HO‐1 were determined by Western blot determined in lung tissue homogenates (A,B). Analysis of the NFκB p65 protein, EPx by immunofluorescence in lung sections (C). ^***^ *P* < .001 at the respective weeks, between the positive asthma groups (young negative, old negative) *vs*. the treatment groups (young asthma, old asthma); ^###^ *P* < .001 between the treatment groups at 6 and 24 w. Data were normalized in order to compare between the effects of antioxidant treatment on the asthma mouse model

The Nrf2‐Keap1‐HO1 pathway and NFκB‐IkB pathway were evaluated after vitamin E treatment in the younger and older asthmatic mice by using Western blot. Data were normalized in order to evaluate the effects of vitamin E. The levels of Nrf2, HO‐1, and IkB were restored; in contrast, the levels of NFκB and Keap1 were decreased after vitamin E treatment in the lung tissues of old asthma mice compared to the young asthmatic mice (Figure 5A,B). In immunofluorescence, we finally found the increased expression of NFκB, and EPx was significantly reduced after vitamin E treatment (Figure 5C).

### 3.5 Ex vivo and in vitro effect of vitamin E in human eosinophils

**FIGURE 6:**
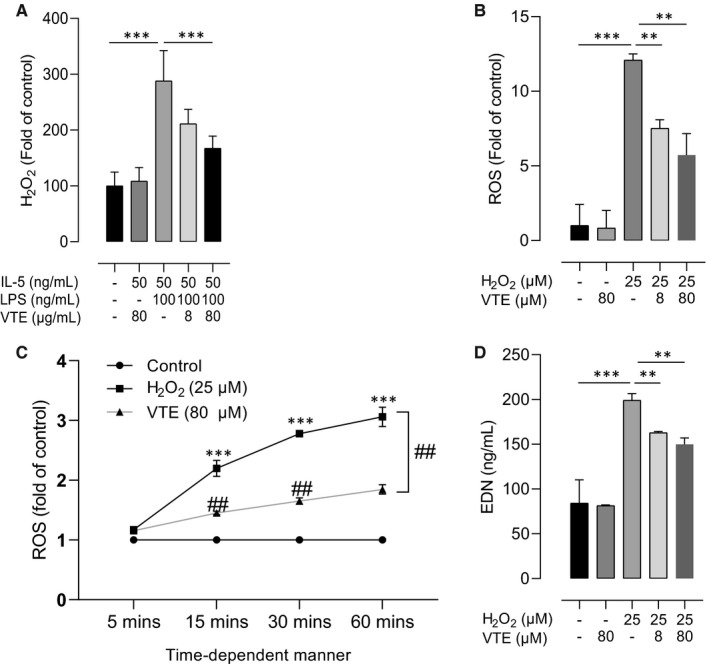
Effects of H_2_O_2_ on human peripheral eosinophil activation. LPS increased the level of H_2_O_2_, which was suppressed by vitamin E treatment in PBEs (A). H_2_O_2_ enhanced ROS release from PBEs in a dose‐dependent manner (B) and time‐dependent manner (C). H_2_O_2_ induced EDN release from PBEs (D). Data are presented as means ± SD. *P < .05, **P < .01, ***P < .001 obtained by Student's *t* test. EDN, eosinophil cationic protein; PBE, peripheral blood eosinophils; PBN, peripheral blood neutrophils; ROS, reactive oxygen species; VTE, vitamin E

Finally, we evaluated oxidant productions and cellular activation after vitamin E treatment on human peripheral eosinophils (PBEs). IL‐5 and LPS‐stimulated PBEs induced the release of H_2_O_2_, which was considered as the main factor to induce ROS formation and EDN release (*P* < .001 for all; Figure 6A‐D), and H_2_O_2_ treatment decreased the expressions of endogenous antioxidants, including SOD and GSH (*P* < .010 for all; Figure 6E‐F). Vitamin E treatment significantly suppressed H_2_O_2_, ROS, and EDN production in dose‐ and time‐dependent manners (Figure S2).

**FIGURE 7:**
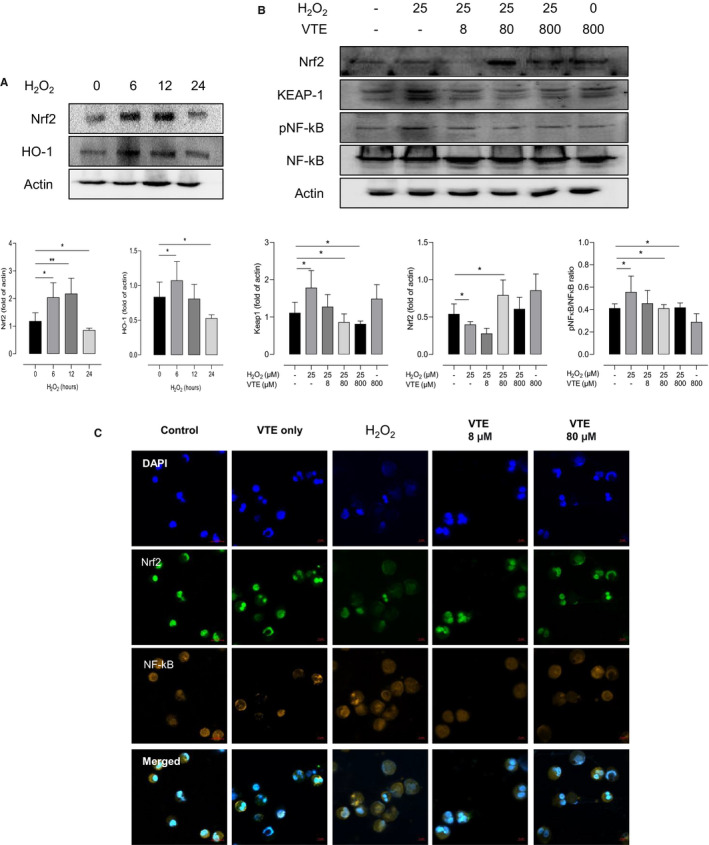
Protective effects of vitamin E against H_2_O_2_ on human peripheral eosinophils. The effects of H_2_O_2_ on the expression of Nrf2 and HO‐1 in the time‐dependent manner (A). H_2_O_2_ increased the expression of NFkB, while decreased the expression of Nrf2, which were reversed by vitamin E treatment evaluated by Western blot (B). The localization of Nrf2 was determined by ICC with anti‐Nrf2 antibody (green) and NFkB (organ) (C). Nuclei was stained by DAPI (blue). Data are presented as means ± SD. *P < .05, **P < .01, ***P < .001 obtained by Student's *t* test. EDN, eosinophil cationic protein; VTE, vitamin E

The expressions of Nrf2 and HO‐1 were increased after 6‐ and 12 hours H_2_O_2_ stimulations; however, these levels were inversely decreased after 24 hours H_2_O_2_ stimulations (*P* < .050 for all, Figure 7A). Importantly, vitamin E treatment showed the restoration of Nrf2 levels on PBEs. Particularly, it could increase the levels of Nrf2 and HO‐1 induced by H_2_O_2_ stimulations, but it decreased NFκB and Keap1 levels (*P* < .001, Figure 7B‐C).

## 4 DISCUSSION

It is well‐known that aging causes a gradual loss of tissue and organ function processes through accumulation of damage caused by ROS.^24^ ROS can be divided into 2 sources: one is the endogenous source of ROS mainly derived from immune cells, such as neutrophils, monocytes, and eosinophils,^2^ and the other is the exogenous source of ROS, which is generated by exposure to the environmental factors that come inside the body and get metabolized into free radicals.^25^ ROS plays a role as defense systems to remove invading pathogens and to maintain redox homeostasis and cellular signal transduction.^2^, ^5^ However, at high concentration, it has negative effects on the oxidative modification of major cellular macromolecules such as lipids, DNA, and proteins.^24^ However, the exact mechanisms of oxidative stress‐induced aging in asthma are still not clear.

Elderly asthma, one of the phenotypes of asthma, is difficult to treat because of steroid resistance, non‐type 2 phenotype, and low atopy rate.^3^, ^4^ They could have significant alterations in pulmonary inflammation and structural changes during development of asthma with age.^26^ Due to these clinical features, elderly asthma has become an important medical burden whose mechanism and novel therapeutic options according to its mechanism are warranted.

In the present study, we found different features of airway inflammation in younger and older asthmatic mice. First of all, the degree of airway inflammation in the BAL and lung tissues was significantly increased in the elderly asthmatic mice compared to the younger asthmatic mice, although there was no difference in AHR. These results are compatible with those of previous studies.^26^, ^27^, ^28^ Furthermore, we found significantly elevated IL‐5 levels, which suggest markedly increased eosinophilia in the older asthmatic mice. IL‐5 can enhance chemokine‐primed ROS production from eosinophils as well,^29^ and increased ROS production is another key feature that contributes to frequent exacerbations and hospitalizations in elderly asthmatics.^30^

Second, we found significant oxidant features which were increased ROS, NFĸB, and Keap1, as well as decreased GSH, SOD, and vitamin E levels in the older asthmatic mice compared to the younger asthmatic mice. ROS have opposite effects against GSH and SOD in the maintenance of body homeostasis.^31^ Previous studies regarding asthma have demonstrated increased ROS levels and declined endogenous antioxidant function in older asthmatic patients compared to younger asthmatic patients.^32^, ^33^ Additionally, we showed reduced Nrf2 levels in the older asthmatic mice compared to the younger asthmatic mice, whereas the expression of p65 NFĸB was shown to be significantly increased in the lung tissues. Nrf2 is a transcriptional factor that controls more than 20 antioxidant genes in the human body.^34^ Under oxidative stress, Nrf2 translocates to the nucleus, binds to *ARE* genes, and leads to reductions in the expression of pro‐inflammatory cytokines.^34^ Oppositely, NFĸB is another transcription factor that regulates the de novo synthesis of pro‐inflammatory mediators; p65 NFĸB, one of the subunits of the NFĸB family, has a transactivation domain and is closely related to various diseases including asthma.^34^ The Nrf2‐HO‐1‐Keap1 pathway and IkB‐NFĸB pathway have opposite effects in the control of inflammation, which suggests that they may play a crucial role in the treatment of NFĸB‐related diseases via suppression or inactivation of Nrf2.^35^

Third, we also found that eosinophilic activation was correlated with increased ROS production. H_2_O_2_‐stimulated PBEs release ROS and EDN, which contributed to airway inflammation in asthma. Eosinophil granulocyte proteins, such as major basic protein (MBP), eosinophil cationic protein (ECP), eosinophil peroxidase (EPO), and eosinophil‐derived neurotoxin (EDN), were well‐known activation markers for type 2 inflammatory diseases,^36^ and the activation status of eosinophils plays an integral role in eosinophilic asthma.^37^ ROS production is a key feature of activated eosinophils, and excessive ROS production can induce cellular death and tissue destruction to release cellular materials.^38^, ^39^ Taken together, increased eosinophilic activation and high IL‐5 and ROS levels could be disease markers, especially in elderly eosinophilic asthma.

Finally, we evaluated the effect of vitamin E in an asthma model. Antioxidants may act as scavengers of oxidants to maintain the biological redox steady state and to protect the body against aging and age‐related diseases via different mechanisms.^40^ In a normal physiological condition, there is a balance between ROS production and endogenous antioxidants including GSH, SOD, and CAT.^14^, ^41^ However, since endogenous antioxidants alone are not sufficient for neutralizing the high‐levels of ROS, they require supplementation of exogenous antioxidants.^41^ Vitamin E was used as one of the most effective antioxidants in inflammatory and age‐related diseases, as previously reported.^40^ For example, α‐tocotrienols protect mouse hippocampal and cortical neuron cell death by regulating the neurodegenerative signaling cascade,^42^ and administration of tocopherols and tocotrienols can also control age‐related arthritis via reducing cytokine production.^43^ In asthma, accumulating evidence reveals that AHR and airway inflammation are significantly decreased after treatment with vitamin E.^44^, ^45^ Additionally, vitamin E can rescue Nrf2 activity which was attenuated in alveolar macrophages during the late phase of IgE‐mediated inflammation in human atopic asthmatics in vivo.^46^ We found different effects of vitamin E administration according to age, which stimulated downregulation of the IkB‐NFκB pathway and EPx expression to reduce ROS production and eosinophil activation. In addition, vitamin E administration restored the dysregulation of the Nrf2‐HO1‐Keap1 pathway in the lung tissues from the older asthmatic mice. We showed that the exposure of H_2_O_2_ for a long time will decrease the endogenous antioxidant defense, including SOD and GSH. Additionally, H_2_O_2_ can decrease the level of Nrf2 by increasing the Keap1‐Nrf2 interaction. As a result, the antioxidant defense will be disrupted, and overproduction of ROS will be induced. Vitamin E can protect cell activation by enhancing endogenous antioxidant concentrations to suppress ROS levels, as well as decrease Keap1 levels to release active Nrf2. All of these effects of vitamin E have also been demonstrated in in vitro studies using PBEs.

There are some limitations to this study. Our model used 6‐week‐old and 24‐week‐old mice as the younger and older groups, respectively, so it could not completely reflect elderly asthmatic subjects. Moreover, since the majority of human asthma cases in the elderly are mainly neutrophilic asthma, our eosinophilic asthma model could not fully reflect the elderly asthma. These limitations can be overcome by using a neutrophilic asthma model and a much older mouse model in further study.

In conclusion, vitamin E may control airway hyperresponsiveness and inflammation in the elderly asthma cases through its antioxidant and inflammatory effects on NFκB and Nrf2 regulation. Further studies are needed to confirm our results.

## CONFLICT OF INTEREST

The authors confirm that there is no conflict of interest.

## AUTHOR CONTRIBUTION

**Quang Luu Quoc:** Data curation (lead); Formal analysis (lead); Investigation (lead); Methodology (lead); Writing‐original draft (lead). **Tra Cao Thi Bich:** Data curation (supporting); Methodology (supporting). **Seo‐Hee Kim:** Data curation (supporting); Methodology (supporting). **Hae‐Sim Park:** Conceptualization (supporting); Funding acquisition (supporting). **Yoo Seob Shin:** Conceptualization (lead); Supervision (lead); Writing‐original draft (supporting).

## Supporting information

Fig S1

Fig S2
