# Supplementary figures and images for: Administration of vitamin E attenuates airway inflammation through restoration of Nrf2 in a mouse model of asthma
Source: J Cell Mol Med. 2021 Jun 4;25(14):6721–32. doi: 10.1111/jcmm.16675 (PMC8278095; doi:10.1111/jcmm.16675)

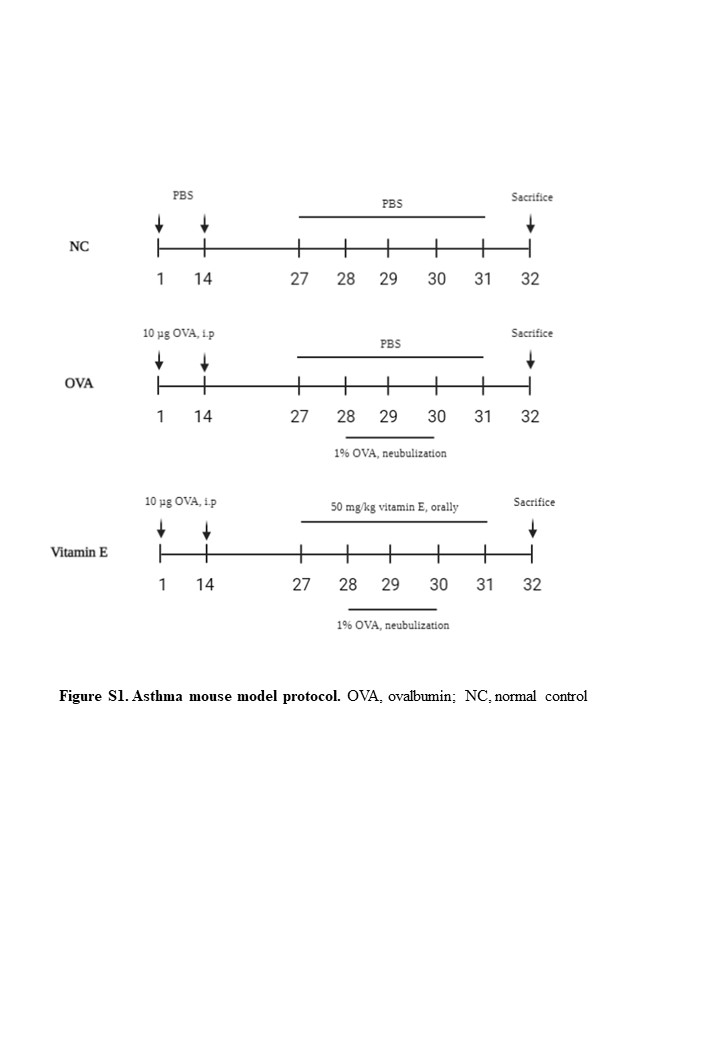

Supplement: Supplementary file 1 — Fig S1 [file JCMM-25-6721-s002.jpg]

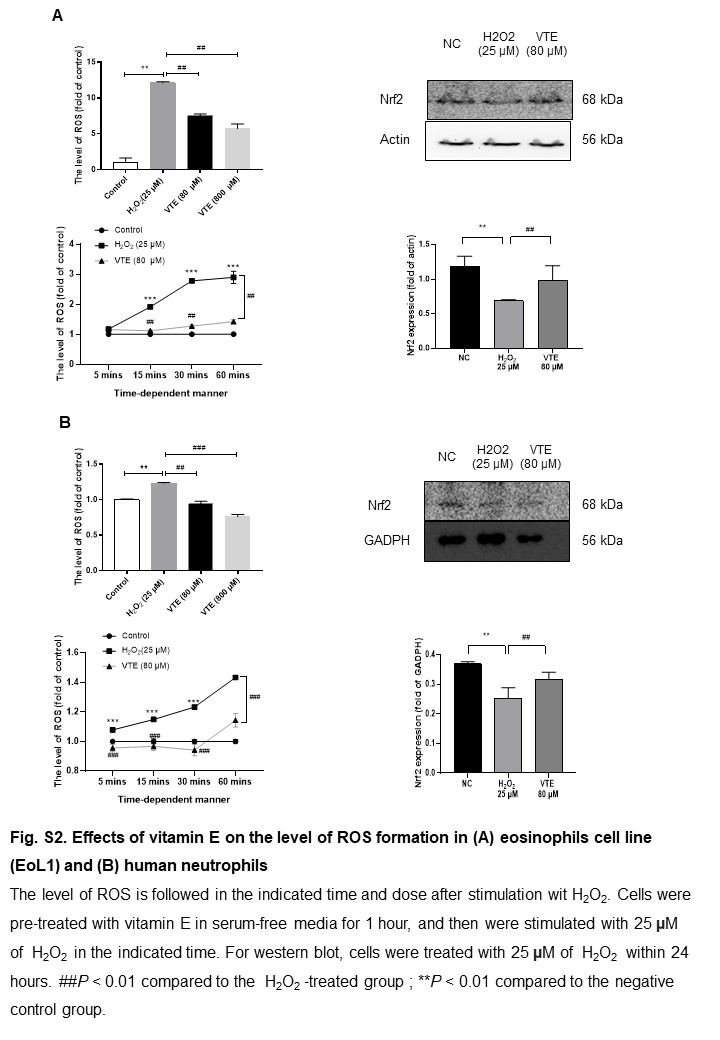

Supplement: Supplementary file 2 — Fig S2 [file JCMM-25-6721-s001.jpg]
